# Identification of *Bordetella bronchiseptica* in the throat and nose of dogs and cats by PCR

**DOI:** 10.22099/mbrc.2022.43873.1755

**Published:** 2022

**Authors:** Mohammad Tabatabaei, Hamid Reza Rohani

**Affiliations:** Department of Pathobiology, Faculty of Veterinary Medicine, Shiraz University, Shiraz, Iran

**Keywords:** Bordetella bronchiseptica, Cats, Dogs, PCR

## Abstract

*B.bronchiseptica* is pathogenic for some domestic and wild animals. Due to the importance of this bacterium, its presence in dogs and cats has been investigated using PCR. Pharyngeal and nasal swabs were taken from 135 dogs and 42 cats. Based on the PCR performed on the dogs' samples, in 25/63 (39.68%) pharyngeal samples and 20/59 (33.89%) nasal samples DNA of *B. bronchiseptica* detected. On the other hand, according to the PCR performed on the cats' samples, in 9/23 (39.13%) pharyngeal samples and 319 (15.78%) nasal samples DNA of *B. bronchiseptica *was existed. According to the present study, the rate of *B. bronchiseptica* infection is high among dogs and cats in Iran. Also, due to the fact that the prevalence of this bacterium among pets animals is not exactly known in Iran, necessary measures should be taken for rapid diagnosis and treatment and proper control of the infection.

## INTRODUCTION

*B.bronchiseptica* colonize respiratory tract of a variety of mammals. This organism is causative agent kennel cough of dogs and suppurative bronchopneumonia in cats [1, 2]. The expression and production of various compounds required for attachment, tissue damage, and possibly changes in the host's defense system by bordetella cause its colonization in the respiratory system and the occurrence of disease [3, 4]. In recent years, the population of dogs and cats as pets and sometimes entertainment for children has increased in human societies. Dogs and cats suffering from most diseases only cause discomfort to the animals themselves, but diseases of the respiratory system can also upset the owner of the animal due to the sounds it makes. In addition, coughing causes the spread of infectious agents and contamination of the surrounding environment. Although cough is one of the most important symptoms of bordellosis in dogs, the symptoms of respiratory infections in cats are often non-specific [5].

Kennel cough is one of the important diseases caused by *B. bronchiseptica* in dogs, which is accompanied by dry and continuous coughs [5]. Although a large number of domestic and wild animals are involved in the transmission of *B. bronchiseptica* to humans; the transmission of infection from pet animals to humans is of particular importance. Given the growing trend of keeping pets in Iran and the zoonotic nature of this bacterium, assessing the level of infection in domestic dogs and cats that are in direct contact with humans can be very important. 

For detection of bordetella infection in animals, many conventional bacteriological, immunological and molecular tools and methods have been used in recent years. Positive cultures of nasal swabs, pharyngeal swabs, and tracheal lavage fluid are required for definitive diagnosis of *B. bronchiseptica* infection [6]. However, Due to being fastidious, slow growth rate and contamination of clinical specimens, it is difficult to diagnose bordetella infection [7, 8]. The polymerase chain reaction (PCR)-based method can be particularly useful for analysis of different samples and provides a platform capable of rapid screening of samples for trace levels of bordetella DNA. In this study, PCR method was used for *Fla* gene locus of *B. bronchiseptica* [9].

## MATERIALS AND METHODS

Pharyngeal and nasal swabs were taken from 135 dogs (76 pharyngeal and 59 nasal samples) and 42 cats (23 pharyngeal and 19 nasal samples) of different ages, sexes, and breeds that were normally admitted to the Faculty of Veterinary Medicine training clinic. Sterile cotton-tip swabs were rubbed on the throat and nasopharyngeal regions, put in the tube containing brain heart infusion (BHI) broth (Merck, Germany), and transferred to the laboratory of microbiology, Faculty of Veterinary Medicine under cold condition. Then, swabs in BHI broth were vortexed and incubated at 37°C for 24 hours as enriched cultures. *B. bronchiseptica  *(PTCC 1025) as positive control was purchased from the Iranian Research Organization for Science and Technology (IROST).

500 μl of enriched culture samples were used for DNA extraction using a DNA extraction kit (Cinnagen, Iran) according to the manufactured description. The PCR reaction mixture contained 12.5 µl Master mix (Amplicon Denmark), 1.5 µl of each *ﬂa* gene-specific primers [9], 3 µl template DNA solutions and 6.5 µl deionized water were prepared. After initial denaturation (94°C/3 min), PCR amplifications were done for 35 cycles in a thermocycler (Analytic Jena, Germany), with a denaturation temperature of 94°C lasting for 1 minute, annealing at 58°C lasting for 1 minute and extended for 1 more minute at 72°C, continuing for 7 minutes at 72°C as the final extension. The purified DNA of *B. bronchiseptica *(ATCC 4617) and sterile deionized H_2_O were used as positive and negative controls, respectively. Then the PCR product evaluated by electrophoresis in 2% (w/v) agarose gel (Agarose I; Cinnagen, Iran) stained with safe stain. 

## RESULTS AND DISCUSSION

**Figure 1:**
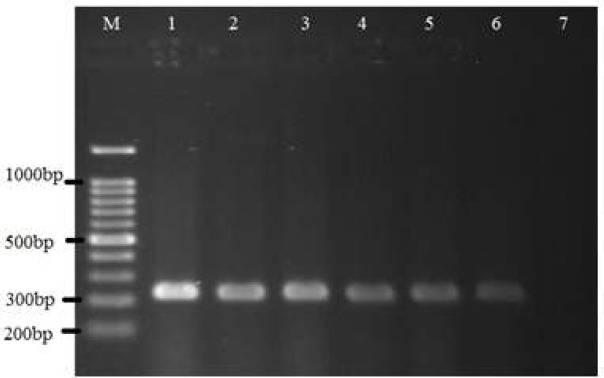
PCR results for presence of *B. bronchiseptica *DNA in different samples. Lane M: 100 bp DNA marker, Lane 1: Positive control, Lanes 2-6: Some positive samples (amplicon size 237 bp), Lane7: Negative control

Based on PCR performed on dogs' pharyngeal and nasal samples, 25 out of 63 (39.68%) pharyngeal samples and 20 out of 59 (33.89%) nasal samples were positive for *B. bronchiseptica *DNA. Also, on cats' nose and pharynx samples, 9 out of 23 (39.13%) pharyngeal samples and 3 out of 19 (15.78%) nasal samples showed positive results for *B. bronchiseptica*  DNA (Fig. 1).

*B.bronchiseptica *as respiratory pathogens colonize the respiratory tract of humans and animals and can cause infections with different signs [10, 11]. *B. bronchiseptica*is considered as one of the main causes of respiratory infections in dogs and cats. Diagnosis of *B. bronchiseptica* infection is usually based on culture and isolation of bacteria. Although these methods are specific, they are time-consuming and sometimes lack the necessary sensitivity. Therefore, in recent years PCR were used to detect infectious agents, including *B. bronchiseptica*. In 1999, Hozbor et al. proposed a PCR method based on a specific intergenomic sequence for the rapid and sensitive diagnosis of *B. bronchiseptica *[9].

According to different study in the UK* B. bronchiseptica *isolated from 3.5 and 11 percent of cats with and without signs of respiratory disease, respectively. Evaluation of different risk factors indicated that large number of cats in a household, and contact with dogs with respiratory tract diseases are very important in *B. bronchiseptica *infection [12, 13].

In Southern Louisiana different methods evaluated for detection of *B. bronchiseptica *in cats without signs of respiratory disease. *B. bronchiseptica *was isolated from 19 out of 614 cats (3.1%) by oropharyngeal swab and 6 out of 614 cats (0.98%) by bronchial lavage. On the other hand, when using an antibody capture ELISA method, 148/614 cats (24.1%) were seropositive for *B. bronchiseptica* [14].

Serological studies in the UK and the Netherlands on different groups of cats show a high prevalence of *B. bronchiseptica *infection (72-79% and 39%) [12]. Also Stifield *et al*. (2012) reported that 20% the dogs infected with *B. bronchiseptica *showed respiratory symptoms [15]. 

Studies by Hoppe (1999) and Register (2001) concluded that PCR was much more sensitive compared to culture, moreover, culture sensitivity could be affected by a variety of factors such as low bacterial count in the nasal cavity, especially in asymptomatic animals [16,17]. In a study in Germany by real-time PCR *B. bronchiseptica *was detected in 78.7% of dogs with respiratory symptoms and 45.6% of healthy dogs. In addition in 52% of dogs with respiratory symptoms, *B. bronchiseptica *was identified as the only cause of respiratory disease [18]. In 2015, Singh et al., reported that DNA extracted from serum samples could be used for molecular detection of bordetella infection in domestic dogs [19].

Study of Coutts et al., in 1996 showed that healthy seropositive queens can be carriers of *B. bronchiseptica*. Although none of the queens showed a detectable amount of *B. bronchiseptica* excretion before delivery, *B. bronchiseptica* were isolated from the queens from 9 days to 12 weeks after parturition. It is possible that various stressors and physiological changes such as parturition and lactation may play a role in bacterial shedding [20].

In 2020, Afi et al., used culture and PCR to diagnose *B. bronchieptica* infection in seemingly healthy domestic and kenelled dogs and dogs with symptoms of respiratory disease. According to the culture results, *B. bronchieptica* were detected in 3.2% of domestic and kenneled dogs with clinical signs of respiratory disease and 6.4% of kenelled dogs without clinical signs. While the culture result of none of the healthy domestic dogs was positive. On the other hand, PCR results for the presence of *B. bronchieptica* in 16.1 and 9.6% of domestic dogs with respiratory disease symptoms and no clinical symptoms, respectively, and 22.5 and 16.1% of kenneled dogs with respiratory symptoms and seemingly healthy dogs were positive [21].

Also, in a study conducted by Isvand et al. (2021) using PCR on 50 samples of suspected to having canine distemper symptoms and 50 seemingly healthy dogs, they were found that the PCR result was positive for 20 samples of dogs suspected to having distemper symptoms and one sample of seemingly healthy dogs for the presence of bordetella DNA [22]. In studies by Afi et al. (2020) and Isvand et al. (2021) presence of *B. bronchiseptica* showed by culture and PCR in pet and kenelled dogs with and without clinical signs of respiratory diseases in Iran [21,22]. These data established that PCR is a reliable method for the identification of *B. bronchiseptica*.

But to the authors’ knowledge, there are no any studies about the prevalence of *B. bronchiseptica *in cats in Iran. Therefore, our study aimed to investigate the occurrence of *B. bronchiseptica *in dogs and cats**.** The true prevalence of *B. bronchiseptica* is likely higher than what is presented here due to the fact that, a variety of factors can lead to bacterial detection failure. The present study shows a significant level of *B. bronchiseptica* infection in domestic dogs and cats so it is recommended that sensitive groups avoid contact with dogs and cats. 

## Conflict of Interest:

On behalf of all authors, the corresponding author states that there is no conflict of interest.

## References

[1] Speakman AJ, Dawson S, Binns SH, Gaskell CJ, Hart CA, Gaskell RM (2008). Bordetella bronchiseptica infection in the cat. J Small Anim Pract.

[2] Goodnow RA (1980). Biology of Bordetella bronchiseptica. Microbiol Rev.

[3] Urisu A, Cowell JL, Manclark CR (1986). Filamentous hemagglutininhas a major role in mediating adherence of Bordetella pertussis to human WiDr cells. Infect Immun.

[4] Everest P, Li J, Douce G, Charles I, De Azavedo J, Chatfield S, Dougan G, Roberts M (1996). Role of Bordetella pertussis P 69/pertactin protein and the P 69 RGD motif in adherence to and invasion of mammalian cells. Microbiology.

[5] Keil DJ, Fenwick B (1998). Role of Bordetella bronchiseptica in infectious tracheobronchitis in dogs. J Am Vet Med Assoc.

[6] Quinn PJ, Markey BK, Leonard FC, Hartigan P, Fanning S, Fitzpatrick ES (2011). Veterinary Microbiology and Microbial Disease. Wiley-Blakwell.

[7] Hoskins JD (1999). Feline respiratory diseases. Vet Clin North Am Small Anim Pract.

[8] Bhardwaj M, Singh BR, Vadhana P (2013). Bordetella bronchiseptica infection and kennel cough in dogs. Adv Anim Vet Sci.

[9] Hozbor D, Fouque F, Guiso N (1999). Detection of Bordetella bronchiseptica by polymerase chain reaction. Res Microbiol.

[10] Gerlach G, von Wintzingerode F, Middendorf B, Gross R (2001). Evolutionary trends in the genus Bordetella. Microbes Infect.

[11] Diavatopoulos DA, Cummings CA, Schouls LM, Brinig MM, Relman DA, Mooi FR (2005). Bordetella pertussis, the causative agent of whooping cough, evolved from a distinct, human associated lineage of B. bronchiseptica. PLoS Pathog.

[12] McArdle HC, Dawson S, Coutts AI, Bennett M, Hart CA, Ryvar R, Gaskell RM (1994). Seroprevalence and isolation rate of Bordetella bronchiseptica in cats in the UK. Vet Rec.

[13] Speakman AJ, Dawson S, Binns SH, Gaskell CJ, Hart CA, Gaskell RM (2008). Bordetella bronchiseptica infection in the cat. J Small Anim Pract.

[14] Hoskins JD, Williams J, Roy AF, Peters JC, McDonough P (1998). Isolation and characterization of Bordetella bronchiseptica from cats in Southern Louisiana. Vet Immunol Immunopatho.

[15] Steinfeld A, Prenger-Beringhoff E, Bauer N, Weiß R, Morit A (2012). [Bakterien isolate ausdemunteren respiration strakt von erkranktenhunden und derenaktuelleresistenz situation (Bacterial susceptibility testings of the lower airways of diseased dogs)]. Tierarztliche Praxis.

[16] Hoppe JE, Murray, PR, Baron, EJ (1999). Bordetella. Manual of clinical microbiology.

[17] Register KB (2001). Novel genetic and phenotypic heterogeneity in Bordetella bronchiseptica pertactin. Infect Immun.

[18] Schulz BS, Kurz S, Weber K, Balzer HJ, Hartmann K (2014). Detection of respiratory viruses and Bordetella bronchiseptica in dogs with acute respiratory tract infections. Vet J.

[19] Singh PL, Singh BR, Bhardwaj M, Vadhana P, Sinha DK, Boby N, Agrawal RK, Pawde AM (2015). Detection of Bordetella bronchiseptica in serum of apparently healthy and clinically sick pet dogs. Advan Anim Vet Sci.

[20] Coutts AJ, Dawson S, Binns S, Hart CA, Gaskell CJ, Gaskell RM (1996). Studies on natural transmission of Bordetella bronchiseptica in cats. Vet Microbiol.

[21] Afi F, Jamshidi S, Bokaie S, Nayeri Fasayi B, Ashrafi Tamay I, Delrobaei M, Zahraei Salehi T (2020). Detection of Bordetella bronchiseptica in oropharynx region of pet and kenneled dogs by PCR and culture and evaluation of antibiotic susceptibility of the isolates. J Vet Res.

[22] Isvand M, Mokhtari A, Esmailnejad A (2021). Genomic and immunological identification of canine distemper virus (Cdv) and investigation of coinfection with Bordetella bronch-iseptica among dogs in Iran. Jentashapir J Cell Mol Biol.

